# Small Animal In Situ Drug Delivery Effects via Transdermal Microneedles Array versus Intravenous Injection: A Pilot Observation Based on Photoacoustic Tomography

**DOI:** 10.3390/pharmaceutics14122689

**Published:** 2022-12-01

**Authors:** Yingying Zhou, Xiazi Huang, Jiyu Li, Ting Zhu, Weiran Pang, Larry Chow, Liming Nie, Lei Sun, Puxiang Lai

**Affiliations:** 1 Department of Biomedical Engineering, The Hong Kong Polytechnic University, Hong Kong SAR, China; 2 The Hong Kong Polytechnic University Shenzhen Research Institute, Shenzhen 515100, China; 3 Department of Biomedical Engineering, City University of Hong Kong, Hong Kong SAR, China; 4 Department of Applied Biology & Chemistry Technology, The Hong Kong Polytechnic University, Hong Kong SAR, China; 5 Research Center of Medical Sciences, Guangdong Provincial People’s Hospital, Guangdong Academy of Medical Sciences, Guangzhou 510000, China; 6 Photonics Research Institute, The Hong Kong Polytechnic University, Hong Kong SAR, China

**Keywords:** photoacoustic computed tomography, transdermal drug delivery, microneedles array, injection

## Abstract

Intravenous injection is a rapid, low-cost, and direct method that is commonly used to deliver multifarious biotherapeutics and vaccines. However, intravenous injection often causes trauma or tissue injury that requires professional operation. Transdermal drug delivery overcomes the aforementioned defects, and the microneedles (MNs) array is one of the most promising transdermal drug delivery platforms. Timely, precise, and non-invasive monitoring and evaluation of the effects of MNs in transdermal administration is significant to the research of drug efficiency response to specific diseases. In this sense, photoacoustic computed tomography (PACT), which provides wavelength-selective and deep-penetrating optical contrast, could be a promising imaging tool for in situ evaluation of the treatment effects. In this work, we propose the use of PACT to non-invasively assess the effects of real-time drug delivery in glioma tumors through transdermal administration with degradable indocyanine green-loaded hyaluronic acid MNs (ICG-HA-MNs). The outcome is systematically and quantitatively compared with that via intravenous injection. It is found that the photoacoustic signals of ICG in the tumor site express a faster elevation and shorter duration time in the intravenous injection group; by contrast, the photoacoustic signals demonstrate a lower intensity but prolonged duration time in the MNs group. The observed phenomenon indicates faster response but shorter drug duration for intravenous injection, which is in contrast with the lower loading but prolonged performance for transdermal drug delivery with MNs. These results exhibit good consistency with the earlier, common-sense findings reported from other aspects, confirming that PACT can serve as a potential imaging tool to precisely, non-invasively, and quickly evaluate in situ drug delivery effects and provide constructive guidance for the design and fabrication of microneedles.

## 1. Introduction

As one of the most common drug delivery methods, intravenous injection provides a fast, cost-effective, and direct way to deliver almost any biotherapeutic drugs and vaccines into the body to realize the goal of treatment. Injection syringe, however, usually needs to be operated by healthcare professionals, which is not convenient for home care. Moreover, the pain and potential risk of wound infection associated with the injection of syringe lead to further inaccessibility to certain patients, especially kids [1,2]. Transdermal drug delivery overcomes the disadvantages of intravenous administration, offering various merits such as better compliance and reduced systemic drug interactions [3]. Microneedles (MNs), a promising minimally invasive transdermal drug delivery method, have drawn significant interest and attention in biomedicine in recent years [4,5]. MNs, in general, are fabricated with hundreds of needles of micrometer length to puncture through the stratum corneum barrier to deliver drugs; they are painless, noninvasive or minimally invasive, and convenient to operate [6]. Among diverse types of MNs, polymer MNs are one of the most popular branches due to their satisfactory properties in biocompatibility, biodegradability, nontoxicity, controllable dosage, and easy fabrication [7,8].

As a symbolic drug delivery method, timely monitoring and evaluation of the administration effects of MNs are profitable and worthwhile in appraising the drug performance in specific diseases. It is also constructive to guide further renovation of MNs fabrication. Current techniques to evaluate the drug administration effects of deep-tissue diseases, such as brain tumors [9] and diabetes [10,11], mainly rely on blood tests and bioluminescence imaging, which, however, can be cumbersome and painful for blood tests or unstable for bioluminescence imaging based on the metabolism circulation time and quantity of luciferase substrate [12]. Therefore, imaging technologies that can provide real-time, noninvasive, precise, and in vivo monitoring and analysis are highly desired for evaluating the effects of drug delivery via MNs.

Photoacoustic (PA) tomography is a hybrid imaging technique that uses light for signal excitation and ultrasound for signal detection, combining the advantages of optical absorption contrast and ultrasonic spatial resolution [13,14,15,16,17,18]. Photoacoustic tomography can be roughly divided into two categories, mainly based on the achievable spatial resolutions and the preference application fields—photoacoustic microscopy (PAM) and photoacoustic computed tomography (PACT) [19,20]. PAM exhibits higher resolution yet shallower penetration depth, which has been demonstrated to be attractive for various methods of structural, functional, and molecular microscopic imaging [21,22,23,24]. In contrast, PACT is widely employed in animal and clinical applications with a broader and deeper field of view at a lower resolution [25,26,27]. Benefiting from the wavelength-dependent absorption spectrum for different tissue components, photoacoustic imaging can detect specific targets via endogenous or exogenous absorption contrasts, which can serve as a valuable tool to track the delivery of drugs and further evaluate their effects [28,29]. Monthanchery et al. presented the use of optical resolution PAM to characterize the transdermal delivery of nanoparticles using microneedles, but is still limited in superficial skin [30].

In this study, we propose the use of PACT to precisely and non-invasively monitor the drug delivery effects of transdermal administration by degradable indocyanine green (ICG)-loaded hyaluronic acid MNs (ICG-HA-MNs) in deep mouse glioma models. The drug delivery effects are further systematically and quantitatively compared with intravenous injection. With PACT, signals originating from ICG can be sensitively detected in the tumor region after tail vein injection or MNs puncturing the skin surface. Experimental results indicate that intravenous injection could bring a faster drug-responsive effect but shorter drug duration performance. In contrast, the tumor regions of mice in the MNs group demonstrate a lower drug signal intensity yet prolonged drug lasting time, corresponding to limited drug loading and controllable drug release characteristics, respectively. The efficiency of transdermal administration by MNs can be further improved if more ICG can be loaded. Although many aspects can be further investigated in the next phase, to the best of our knowledge, this pilot study is the first to try to compare the drug delivery effects between intravenous injection and transdermal drug delivery (by MNs) through a quantitative imaging aspect by PACT, which overcomes the deficiencies of conventional monitoring methods. The results suggest that PACT can be used as a potential in situ imaging modality to evaluate the effects of drug delivery on small animals and provide constructive guidance for the design and fabrication of MNs.

## 2. Materials and Methods

### 2.1. Cells Culturing and Preparation

Luciferase-tagged U87 glioma cells were cultured with high glucose Dulbecco’s modified Eagle’s medium (DMEM) supplemented with 10% fetal bovine serum (FBS) and 1% penicillin-streptomycin [31]. After three cell passage times, the cells in good conditions were centrifugated and washed twice with the phosphate-buffered saline (PBS) to get the cell pellet.

### 2.2. U87 Xenograft Tumor Model

Twelve female Balb/c nude mice were anesthetized by a mixture of ketamine (100 mg/kg) and xylazine (10 mg/kg). With the help of a stereotaxic instrument, the Luciferase-tagged U87 cells (1 × 10^6^ cells in a volume of 5 μL) were inoculated into the right caudate nucleus of the mice to establish the U87 xenograft tumor model [32]. All procedures in the animal experiments were approved by the animal ethical committee of the Hong Kong Polytechnic University. Bioluminescence imaging using the Perkin-Elmer IVIS Lumina Series III system was conducted approximately 14 days after cell implantation to ensure the successful creation of glioma tumor models [33]. The U87 tumor model would express fluorescence signals only a few minutes after the injection of luciferin substrate.

### 2.3. Materials and the Fabrication Process of ICG Microneedles Array

Indocyanine green (ICG), the sole infrared-contrast agent approved by the Food and Drug Administration (USA) for clinical applications, was used to serve as the drug to be delivered and monitored. The ICG (dye content 90%) was purchased from Beijing J&K Scientific Co., Ltd. (Beijing, China). Hyaluronic acid (HA), widely adopted to fabricate dissolving microneedles for transdermal drug delivery, was chosen in this study [34,35,36]. The sodium hyaluronic (M_w_ < 10,000) was supplied by Bloomage Biotech (Jinan, China).

**Figure 1:**
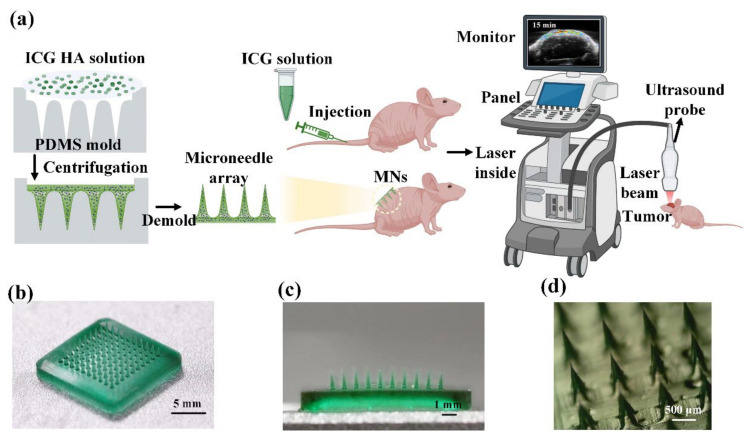
(**a**) Schematic of the fabrication process of ICG-HA-MNs and an illustration of the experimental procedure. (**b**) Photography of ICG-HA-MNs patch in full view. (**c**) Cross-sectional view of the ICG-HA-MNs patch. (**d**) Stereomicroscopy image of the ICG-HA-MNs.

The ICG-HA-MNs were fabricated via two casting methods [37]. Firstly, the MNs metal master mold containing MNs arranged in 10 × 10 arrays, with a needle height of 900 μm and base area of 0.09 mm^2^ (300 μm × 300 μm), was immersed into Polydimethylsiloxane (PDMS) solution. The PDMS solution with MNs mold was put into the oven at a temperature of 65–70 centigrade for 3 h to dry after being degassed. The MNs metal master was removed after the PDMS was cured, obtaining the PDMS mold as shown in Figure 1a. A total of 2 mL casting solution mixed with ICG solution (1 mg/mL) and HA solution (1 g/mL) at a volume ratio of 1:1 and was then cast into the PDMS mold which was placed in a 50 mL corning tube. The final mixed solution was spread on the surface of the PDMS mold. The corning tube containing the PDMS mold and drug-loaded solution was centrifugated at 4000 rpm for 10 min to fill the cavities in the PDMS mold. Subsequently, the PDMS mold filled with ICG-loaded solution was taken out and dried at room temperature for 24 h. The microneedles array could be easily demolded in the next day, and the microneedles array was finally ready.

### 2.4. Optical and Mechanical Characterizations of MNs

Macro- and micro-optical images of the ICG-HA-MNs were obtained by digital camera and microscopy, respectively. To ensure the mechanical property of the MNs, a patch of fresh porcine skin was vertically punctured by the ICG-HA-MNs for 5 min, and then taken for tissue fixation and histological examination. Skin sections were attached on a glass slide, and the intactness of the stratum corneum was observed under microscopy.

### 2.5. PACT Monitoring and Evaluation of the Drug Delivery Effects

The PA platform used for this work was FUJIFILM Visual Vevo LAZR multi-modality imaging system, equipped with a broadband ultrasound frequency transducer (Vevo LAZR LZ250, center frequency: 21 MHz, bandwidth:13–24 MHz). The wavelengths used in this study were 680, 695, 732, 882, and 924 nm. The energy used for each wavelength is about 50 mJ. Using multiple wavelengths can help to reduce the background noise and improve the signal-to-noise ratio as well as image contrasts. In the tumor site, there are many blood vessels (hemoglobin and non-hemoglobin), which also absorb light of a wide range of wavelengths and produce strong photoacoustic signals. In the case of blood signal interference by using only one absorption wavelength (ICG absorption), the induction of multiple wavelengths and spectral unmixing can help to distinguish different contrasts, so that the ICG signal at the tumor location can be more accurately acquired and demonstrated. The ICG has high absorption in the 700–900 nm wavelength range, so we chose three wavelengths in this range as the trigger of ICG, which shows relatively weak absorption for hemoglobin at the same time. In addition, 680 nm and 924 nm were also induced to specifically target oxyhemoglobin and deoxyhemoglobin. Finally, the background signals from the blood can be extracted through spectral unmixing using the VevoLAB software. The “spectral unmixing” is a system built-in data process function provided by the VevoLAB software. The Vevo LAZR system has already calibrated absorption spectrum of various contrasts, including oxyhemoglobin, deoxyhemoglobin, ICG, etc. Hence, the photoacoustic signals of ICG can be separated and protruded based on the differences in the absorption spectra between different contrasts.

Before the experiments, the mice were divided into two groups (n = 3/group) and anesthetized with isoflurane. In the injection group, ICG solutions (200 nmol in 0.15 mL for each mouse) were injected into the tail veins. In the MNs group, two patches of ICG-HA-MNs were punctured into the back skin of mice for 5 min. Well-prepared mice were placed on the heating pad and monitored by the abovementioned PA/US dual-mode platform following the timeline. Given the high metabolism rate of the ICG reported in earlier research [38], the timeline chosen in this study was pre experiment, 15 min, 30 min, 1 h, 2 h, 3 h, 4 h, and 24 h post drug delivery.

### 2.6. Statistical Analysis

The student’s two-tailed, unpaired *t*-test was adopted to compare the two groups with the corresponding *p*-value. Each asterisk in the plot denotes a significant difference between the two data groups (*p* < 0.05) unless additionally specified in the figure caption. Standard errors were noted, taking into the account of the subject-to-subject variations, and represented as error bars in the quantitative result figures.

## 3. Results

### 3.1. Characterization of MNs

As shown in Figure 1b, 10 × 10 cone-shaped microneedles are organized and uniformly fabricated on the substrate. The whole patch expresses a uniform dark green color, reflecting the color of the ICG solution. Figure 1c shows the cross-section of the ICG-HA-MNs patch. The uniform green color of the needles indicates that the ICG was loaded into the HA microneedles homogenously. The microneedles’ average height and base diameters were 900 and 300 μm, respectively. More information about the microneedle tips can be observed in Figure 1d, where the sharp feature helps to puncture the skin more easily.

### 3.2. Skin Penetration Test

**Figure 2:**
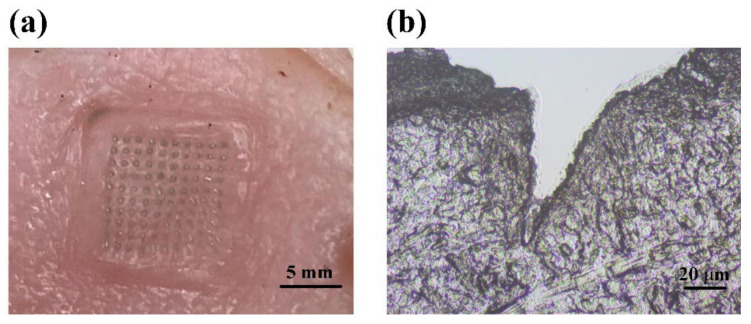
(**a**) Photography of MNs punctured porcine skin. (**b**) Histological slice of the porcine skin along the cross-section in the punctured site, showing the trace of microneedle insertion into the tissue sample.

After being punctured by the ICG-HA-MNs for around 5 min, the substrate of the MNs patch was removed from the porcine skin. The tips of the MNs patch were dissolved in the skin, as shown in Figure 2a. The surface of the porcine skin resembles the regular and uniform shape of the MNs. The light green ICG dot array on the skin surface fit well with the arrangements of the microneedles. Figure 2b shows the histological view of the cross-section of the punctured porcine skin. A hole with a depth of approximately 80 μm created by the microneedles can be observed, with ICG dissolved inside. This result indicates that the ICG-HA-MNs possess enough mechanical property to penetrate the stratum corneum to deliver the drugs as the layer thickness of the stratum corneum is around 10–15 μm.

### 3.3. Dynamic Monitoring of the Drug Delivery Effects via Intravenous Injection

**Figure 3:**
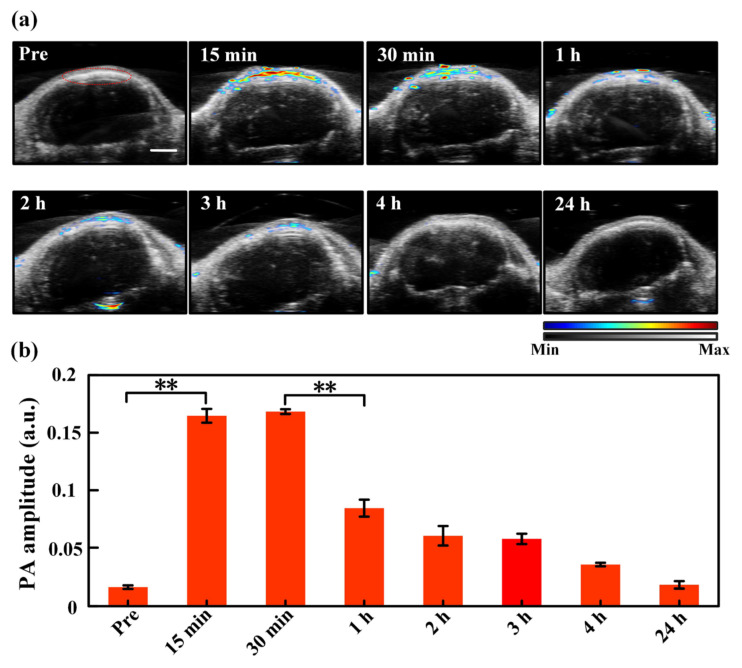
(**a**) Ultrasound (the grey background) and PA (the colored values) images of the tumor region with time pre and post injection. The region encircled by the red curve in the “Pre” picture is the region of interest (ROI) used for data analysis, which is identical for all images acquired at different time moments. The color bar indicates the intensities of PA/US signals. (**b**) Quantitative PA amplitude of ICG signals (spectral unmixing) in the tumor region at different time points after injection. **, *p* < 0.01 (tested by Student’s two-tailed, unpaired *t*-test). The error bars in (**b**) are standard deviations. Scale bar: 2 mm.

With the help of the IVIS bioluminescence detection system, we first checked if the U87 xenograft tumor model was established successfully. The mouse brain tumor regions presented fluorescence signals after luciferin injection, indicating successful animal tumor modeling. Then, the PACT monitoring experiments were conducted. Figure 3a shows the ultrasound (grey background) and PA (colored values) images of the tumor region that change with time. As seen, no ICG signal is observed before injection, and the tumor region expresses strong ICG signals just 15 min post injection. The strong ICG signals sustain for 30 min, which, however, is followed with rapid decrease and even reduce to baseline 4 h post injection. The rapid rise of ICG’s PA signal after the injection is because the intravenously injected ICG directly enters the blood circulation and reaches the tumor site in a short while [39], the sudden drop of the signal is because the ICG that enters the circulation is rapidly metabolized and then excreted [38,40]. The PA signals in the encircled ROIs are selected and calculated to make a quantitative comparison, as illustrated in Figure 3b. It is confirmed that the ROIs manifest strong ICG signals in 15 min post injection with a statistically significant difference compared to the baseline. The ICG signals display a maximum amplitude in 30 min, a rapid drop after 30 min, and then gradual decrease back to the baseline after ~4 h, which is consistent with the trend shown in Figure 3a and previous reported results [38]. Note that the quantitative PA signal intensity in pretreatment is not zero, and it should be the background noise, which is a common phenomenon in photoacoustic imaging [38,41,42].

### 3.4. Dynamic Monitoring of the Transdermal Administration Effects via MNs

**Figure 4:**
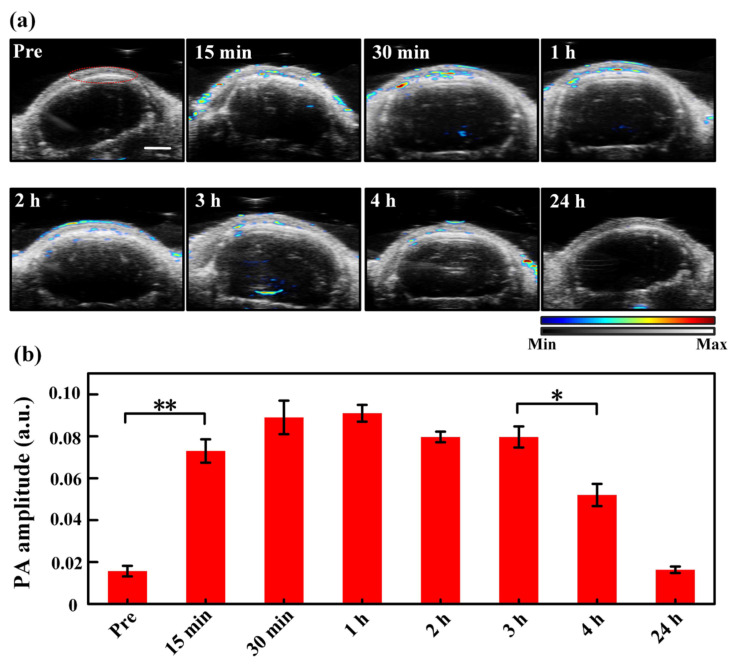
(**a**) Ultrasound (the grey background) and PA (the colored values) images of the tumor region with time pre- and post-MNs puncturing. The region encircled by the red curve in the “Pre” picture is the region of interest (ROI) used for data analysis, which is identical for all images acquired at different time moments. The color bar indicates the intensities of PA/US signals. (**b**) Quantitative PA amplitude of ICG signals (spectral unmixing) in the tumor region at different time points after MNs puncturing. **, *p* < 0.01, *, *p* < 0.05 (tested by Student’s two-tailed, unpaired *t*-test). The error bars in (**b**) are standard deviations. Scale bar: 2 mm.

Figure 4a depicts the ultrasound and PA images of the tumor region with time using the MNs to deliver ICG. As seen, the tumor site shows a clear PA signal from ICG 15 min after MNs puncture, and the strong PA signal lasts about 3 h. PA images acquired 4 h after MNs puncture still demonstrates a significant ICG level. Quantitative PA amplitude analysis of the ROIs circled by the red line in Figure 4a is exhibited in Figure 4b, where the ICG signal in the tumor region elevates to a statistically significant different level 15 min post-MNs puncture as compared to the baseline. The PA signal of ICG rises to its maximum amplitude around 1 h post-MNs puncture, and the high-level PA signal lasts ~3 h. Even 4 h post-MNs puncture, the PA signal remains at more than half of the maximum level. The prolonged and constant drug delivery performance corresponds to the characteristics of transdermal drug delivery with MNs. In the transdermal drug delivery process, the drug passes through the skin at a certain rate and is absorbed into the blood circulation through penetrating through the capillaries. The drug release rate is limited by the rate at which a drug penetrates the capillaries [43,44].

### 3.5. Comparison of the Drug Delivery Effects between Injection and MNs

**Figure 5:**
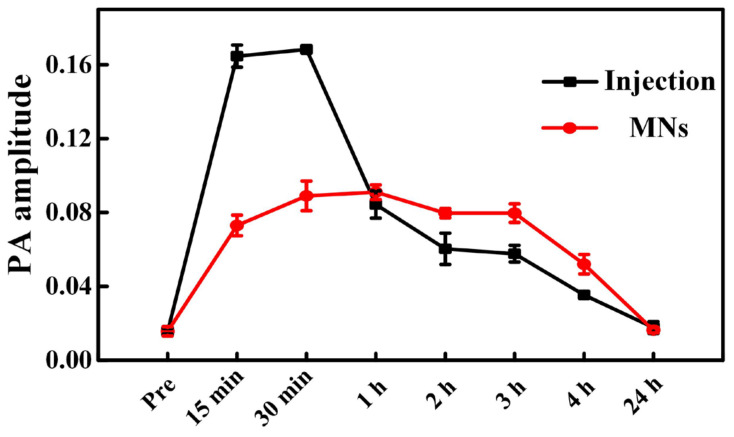
Comparison of the amplitude of PA signals originated from the delivered ICG after injection and transdermal administration of the ICG-HA-MNs.

Figure 5 presents the comparison of the PA signal amplitudes over time following the administration of ICG via the two different drug delivery methods. As seen, the drug signals demonstrate a sharper and higher-level (maximum PA amplitude) elevation within the same time frame by intravenous injection, indicating a faster drug-responsive effect and higher drug-loading performance. That said, the duration of the strong ICG signals in the injection group is shorter than that in the transdermal administration group. Since ICG circulates and metabolizes very quickly in the body, this leads to a sharp drop in the ICG’s PA signal after the intravenous injection [38,40]. In comparison, ICG reveals a lower-level intensity rise yet prolonged durable performance by MNs, suggesting lower drug loading behavior and slower drug release characteristics. This is because ICG passes through the skin at a certain rate and is absorbed into the blood circulation through the capillary to produce the drug effect, which brings long-lasting and constant drug effects [10,45]. Additionally, the ICG signals in both groups increase significantly in 15 min post administration of ICG, suggesting a high metabolism rate and fast responsive property of ICG in the blood circulatory system, which is also consistent with the reported findings [38]. The first time point we chose is 15 min, but this does not mean that no ICG signals were detected before 15 min. It also should be clarified that the observed difference in drug delivery effects between intravenous injection and transdermal administration via MNs may be limited to ICG only; if other micro-dose high-efficiency drugs, such as insulin, are applied, the trend might not be that obvious or even be rather different [10].

## 4. Conclusions

To evaluate the drug delivery efficiency of transdermal administration by MNs and intravenous injection in a precise, non-invasive, and timely manner, we introduced PACT to record the in situ responses from drugs, i.e., PA signals from ICGs, at different time slots and quantitatively compared the ICG signal intensities in the glioma tumor region. It reveals that the in the intravenous injection group, ICG signals exhibit a rapid increase 15 min after tail injection, and the strong PA signals start to decrease rapidly only 30 min post injection. In contrast, in the MNs group, the maximum amplitude of ICG signals is much lower than its peer in the injection group, suggesting less drug loading features of MNs for ICG. However, the ICG signals express a more durable lasting time post-MNs puncture, and the declining time point is much later (around 3 h post MNs) than the injection group. These results suggest that intravenous injection presents a fast drug-responsive property yet a reduced drug duration time, while the transdermal administration by MNs has a relatively low ICG loading efficiency yet prolonged drug release duration. Note that the contrast of PA signals in this study is based on the ICG. Thus, the PA signal amplitudes in the injection group differ significantly from the MNs group (lower ICG load). The differences may not be evident if other micro-dose high-efficiency drugs are used or more ICGs can be loaded in the MNs array. It is exactly this point that indicates that the transdermal administration by MNs should be more suitable for low-dose sensitive drugs of high curing efficiency (not delivery efficiency) in the current stage. In the future, the MNs may also be further improved to carry more drugs to optimize the performance based on requirements. To summarize, this pilot study suggests that the PACT can probably be used as a potential imaging modality to evaluate the drug delivery performance in a precise, non-invasive, and timely manner and provide constructive guidance for the design and fabrication of MNs.

## Data Availability

The data presented in this study are available on request from the corresponding author.
